# 592. Immunogenicity and Safety of High-dose versus Double-dose Quadrivalent Inactivated Influenza Vaccine in Adult Kidney Transplant Recipients: A Randomized Controlled Trial

**DOI:** 10.1093/ofid/ofae631.187

**Published:** 2025-01-29

**Authors:** Thanachote Arayanumas, Kobporn Boonnak, Surasak Kantachuvesiri, Sarinya Boongird, Sasisopin Kiertiburanakul, Jackrapong Bruminhent

**Affiliations:** Faculty of Medicine Ramathibodi Hospital, Mahidol University, Bangkok, Krung Thep, Thailand; Faculty of Medicine Siriraj Hospital, Mahidol University, Bangkok, Krung Thep, Thailand; Faculty of Medicine Ramathibody Hospital, Mahidol University, Bangkok, Krung Thep, Thailand; Faculty of Medicine Ramathibodi Hospital, Mahidol University, Bangkok, Krung Thep, Thailand; Faculty of Medicine Ramathibodi Hospital, Bangkok, Krung Thep, Thailand; Faculty of Medicine Ramathibodi Hospital, Mahidol University, Bangkok, Krung Thep, Thailand

## Abstract

**Background:**

Both high-dose and double-dose inactivated influenza vaccines have been shown to better elicit a greater immune response compared to standard-dose influenza vaccine in solid organ transplant (SOT) recipients. However, a direct comparison between high-dose (HD) and double-dose (DD) quadrivalent inactivated influenza vaccine (QIIV) has not been explored.

**Methods:**

From June to December 2023, we conducted an open-label randomized controlled trial to compare the immunogenicity and safety of the 2023 southern hemisphere HD QIIV at 60 ug (Effluelda, Sanofi) versus double standard-dose QIIV at 30 ug (Fluarix tetra, Sanofi) in adult kidney transplant (KT) recipients. Pre-immunization and 4-week post-immunization sera underwent strain-specific hemagglutination inhibition assays, and safety profiles were assessed.

**Results:**

Eighty-four eligible KT recipients were randomly assigned to receive either HD QIIV or DD QIIV (42 each). Among them, 50 (59.5%) were male, with a mean age (SD) of 46 (11) years old, and 50 (60.7%) participants received kidneys from deceased donors. Common immunosuppressive regimens included mycophenolate mofetil (65.5%), tacrolimus (77.1%), and prednisolone (100%). SC to at least 1 strain was significantly greater in the HD QIIV group (95.2%) compared to the DD QIIV group (69%) (P = 0.006). The SC rate for A/H1N1, A/H3N2, B/Victoria, and B/Yamagata strains was higher in the HD QIIV group compared to the DD QIIV group, with the following percentages: 69.1% vs 52.4%, 52.4% vs 38.1%, 52.4% vs 42.9%, and 61.9% vs 40.5% (P = 0.118, P = 0.188, P = 0.382, and P = 0.049, respectively). In multivariate analysis, SC to at least 1 vaccine strain was significantly associated with the use of HD vaccine (OR 17.59, 95% CI [2.10, 147.22], P=0.008) and receiving the vaccine after 1-year post-KT (OR 34.76; 95% CI [2.59–454.45], P=0.007). There were significantly fewer occurrences of pain at the injection site and muscle ache in the HD QIIV group compared to the DD QIIV group, with the following percentages: 4.8% vs 19.0%, and 0% vs 9.5% (P = 0.040 and P = 0.043, respectively).

**Conclusion:**

HD QIIV could marginally elicit a more pronounced immune response, alongside a comparatively reduced occurrence of adverse reactions, in adult SOT recipients when compared with DD QIIV. TCTR20230510005.

**Disclosures:**

**All Authors**: No reported disclosures

